# A Case of Sickle Cell Beta Thalassemia and Recurrent Septicemia

**DOI:** 10.7759/cureus.87521

**Published:** 2025-07-08

**Authors:** David I LeRoy, Elaine Ognjanovski, Amaani Desai, Anthony V Cook, Rafael Barretto

**Affiliations:** 1 Internal Medicine, Henry Ford Health System - Warren, Warren, USA

**Keywords:** adult sickle cell anemia, hepatic abscess, recurrent sepsis, sepsis, sickle cell beta-thalassemia

## Abstract

Sickle cell anemia and beta-thalassemia are among the most common hemoglobin disorders. They are characterized by abnormal hemoglobin production, leading to ineffective erythropoiesis and severe anemia. Compound forms, such as sickle cell beta-thalassemia (HbS/β-thalassemia), may experience a wide range of complications, including impaired splenic function and increased susceptibility to infections. In this case report, we describe the case of a 57-year-old female patient with sickle cell/beta0-thalassemia (Sß0) with frequent and prolonged hospital admissions for sepsis complicated by recurrent liver abscess requiring multiple procedures for intrahepatic drainage. This patient was found to have an abscess with cholelithiasis, with a multiseptated hypodense collection within the liver. Recurrent liver abscesses are rare and underreported in this population. This case highlights the need for further research to clarify pathophysiology and ultimately improve management.

## Introduction

Hemoglobinopathies, such as sickle cell disease and beta-thalassemia, are inherited disorders that impact hemoglobin structure due to mutations in the β-globin gene [1]. Sickle cell disease is caused by a point mutation that replaces glutamic acid with valine on chromosome 11, leading to structural dysfunction [2]. Beta-thalassemia is characterized by mutations in the β-globin subunit on chromosome 11, leading to reduced or absent globin chain production, causing ineffective erythropoiesis [3, 4]. Beta-thalassemia is often divided by severity, with milder forms being non-transfusion dependent and more severe forms being transfusion dependent [4]. Overlap between these conditions occurs in individuals with a compound heterozygous phenotype for sickle cell and beta-thalassemia (HbSß). This disorder is classified either by an absence of HbA, leading to the phenotype sickle cell/beta0-thalassemia (Sß0), or a decreased production of HbA, leading to the phenotype sickle cell/beta(+)-thalassemia [5]. Sß0 is often associated with a more severe clinical course when compared to its beta+ counterpart [5]. HbSß is a rare phenotype among American sickle cell patients, occurring in <10% of this patient population [6]. These hemoglobinopathies are the second leading cause of surgical asplenia as part of this disease’s treatment course. Additionally, these conditions can lead to secondary hyposplenia, or defective spleen function, as part of natural disease progression, often predisposing this population to infections [7]. Patients with Sß0 often develop secondary hyposplenia early in childhood, resulting in recurrent sepsis [8]. While various abdominal manifestations have been described in both sickle cell and beta-thalassemia patients, recurrent liver abscesses have been rarely described in prior literature [9]. Here we present a case of a 57-year-old female patient with Sß0 who presented with recurrent septicemia due to liver abscess.

## Case presentation

**Figure 1:**
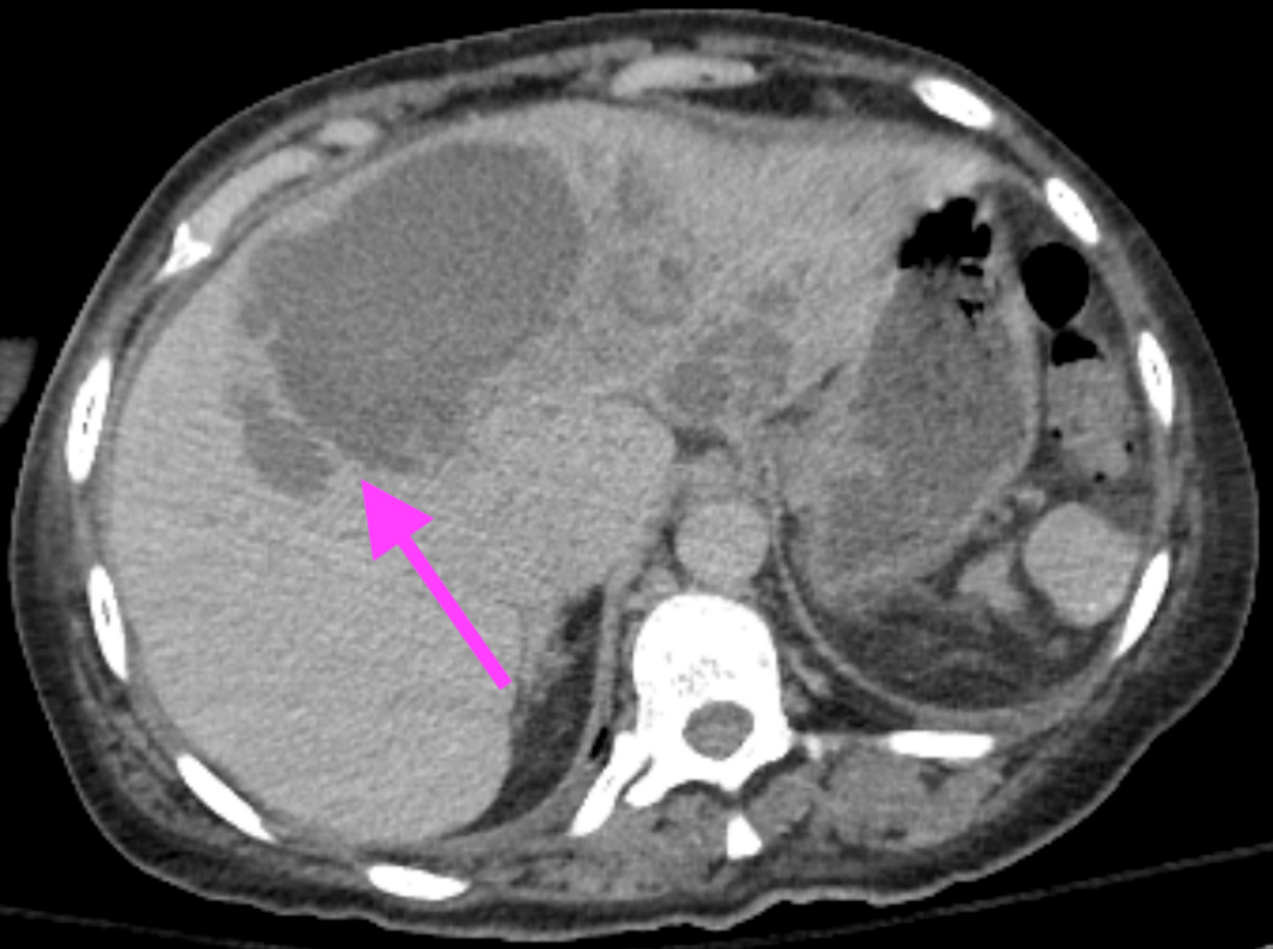
A CT scan of the abdomen and pelvis demonstrated a multiseptated hypodense collection within the liver, indicating an abscess with cholelithiasis. The arrow highlights the intrahepatic abscess.

The patient was a 57-year-old African American female with a past medical history of HbSß, chronic kidney disease, and a history of liver abscess who initially presented to the emergency department because she was feeling unwell. About 3.5 years before this admission, she was diagnosed with a liver abscess and underwent interventional radiology (IR) drainage. However, during this hospitalization, she was found to have septic shock due to lower extremity osteomyelitis with *Klebsiella oxytoca* bacteremia. After completion of antibiotics, the patient was found to have a multiseptated hypodense collection within the liver, indicating an abscess with cholelithiasis seen on the CT scan of the abdomen and pelvis, as shown in Figure 1.

The IR team was consulted and placed a percutaneous intrahepatic drain, with cultures positive for *Enterobacter cloacae* complex. The patient was discharged home on meropenem with a percutaneous intrahepatic drain in place. Three months later, the patient presented again to the hospital and was admitted for septic shock. Pertinent labs for her first and second admissions are shown in Table 1.

**Table 1:** Pertinent laboratory values from the primary admission and admission three months before with reference ranges WBC: white blood cells; Abs retic: absolute reticulocyte; Alk phos: alkaline phosphatase; AST: aspartate transaminase; ALT: alanine transaminase

Pertinent lab	Values three months prior	Primary hospitalization values	Reference range
WBC	31.42 K/mcL	7.18 K/mcL	4.00-11.00 K/mcL
Hemoglobin	4.0 gm/dL	3.9 gm/dL	12.0-16.0 gm/dL
Platelet	273 K/mcL	354 K/mcL	150-400 K/mcL
Abs retic	0.13 Million/mcL	0.11 Million/mcL	-
Reticulocyte	5.27%	7.60%	0.70-1.80%
Bilirubin, total	3.0 mg/dL	1.7 mg/dL	0.0-1.5 mg/dL
Bilirubin, direct	1.5 mg/dL	0.6 mg/dL	0.0-0.4 mg/dL
Alk phos	172 IU/L	231 IU/L	20-130 IU/L
AST	32 unit/L	17 unit/L	0-35 unit/L
ALT	25 unit/L	7 unit/L	0-40 unit/L
Haptoglobin	99 mg/dL	<10 mg/dL	30-200 mg/dL

**Figure 2:**
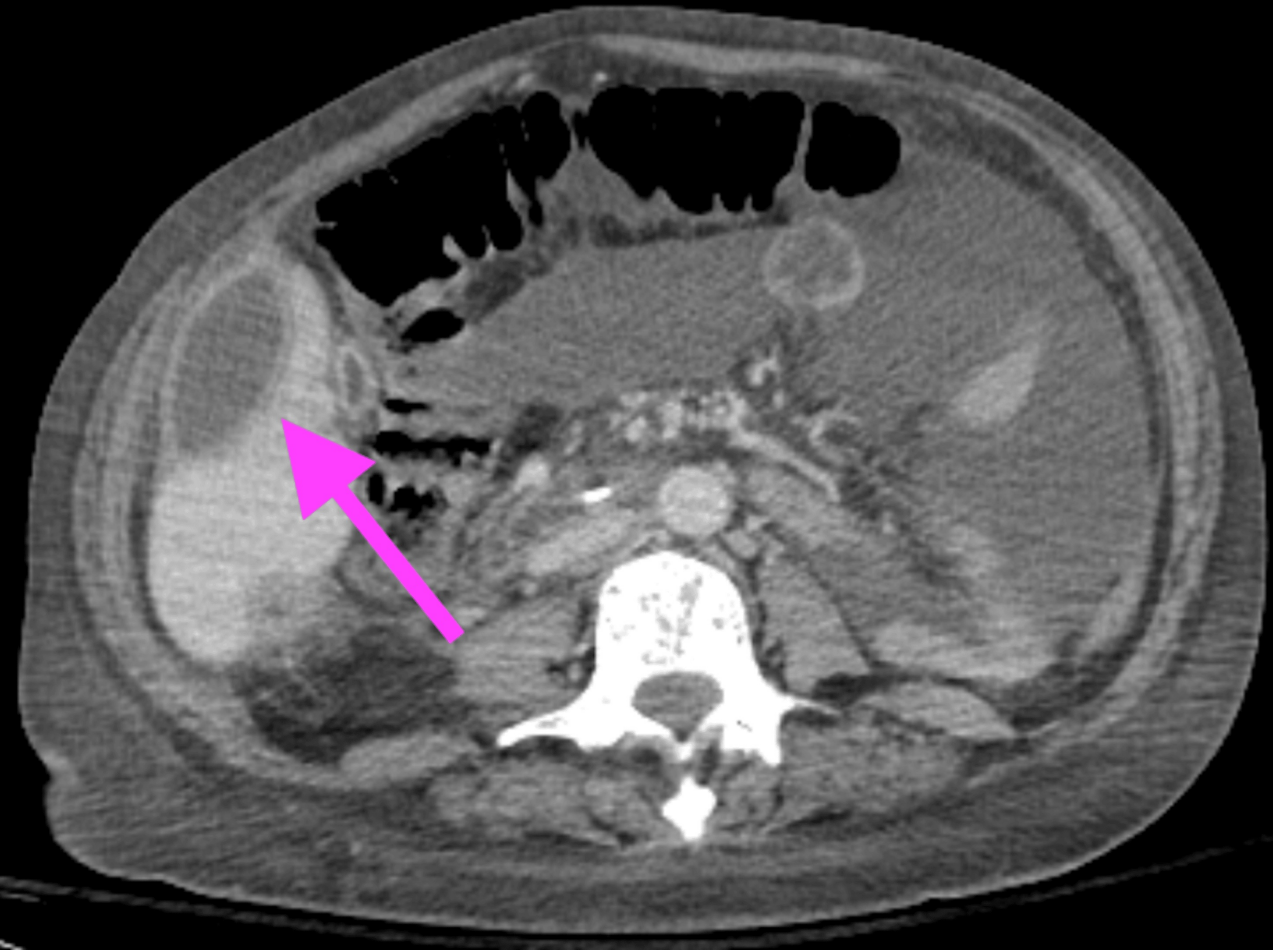
A repeat CT scan of the abdomen and pelvis showed interval development of a rim-enhancing abscess abutting the right hepatic lobe extending inferiorly into the pelvis, measuring 17 cm in diameter, and an interval development of a ring-enhancing abscess in the left subphrenic space, measuring about 7 cm. The arrow highlights intrahepatic abscess.

Repeat imaging at this time showed interval development of a rim-enhancing abscess abutting the right hepatic lobe extending inferiorly into the pelvis measuring 17 cm in diameter, and an interval development of a ring-enhancing abscess in the left subphrenic space, measuring about 7 cm on the CT scan of the abdomen and pelvis, as seen in Figure 2.

During this hospitalization, the patient underwent a paracentesis with IR, removing 1.2 L of clear yellow peritoneal fluid with removal of the intrahepatic drain and reinsertion of the right abdominal pigtail catheter. During this procedure, IR was unable to drain the left subphrenic abscess. Due to the patient's unstable vitals, surgery could not proceed, and the patient was transferred to a tertiary center.

During her final hospitalization six months later, the patient was reported to have been found unresponsive at home by her daughter. She was brought in by emergency medical services (EMS) and found to be hypotensive with labs concerning for severe anemia with hemoglobin of 2.2 gm/dL, hypoglycemia with blood glucose level of 4 mg/dL, potassium of 7.5 mmol/L, creatinine of 3.49 mg/dL, and lactic acid of 13.3 mmol/L. She was also found to have hyperbilirubinemia with a total bilirubin of 7.6 mg/dL, elevated alkaline phosphatase (ALP) of 179 units/L, aspartate aminotransferase (AST) of 899 units/L, and alanine aminotransferase (ALT) of 127 units/L, concerning for hemolysis versus shock liver. All pertinent labs are shown in Table 2.

**Table 2:** Pertinent laboratory values from the final admission WBC: white blood cells; Abs retic: absolute reticulocyte; Alk phos: alkaline phosphatase; AST: aspartate transaminase; ALT: alanine transaminase

Pertinent labs	Final admission	Reference range
WBC	36.65 K/mcL	4.00-11.00 K/mcL
Hemoglobin	2.2 gm/dL	12.0-16.0 gm/dL
Platelet	84 K/mcL	150-400 K/mcL
Abs retics	0.02 Million/mcL	-
Reticulocyte	15.75%	0.70-1.80%
Random glucose	4 mg/dL	70-200 mg/dL
Creatinine	3.49 mg/dL	0.70-1.20 mg/dL
Potassium	7.5 mmol/L	3.5-5.2 mmol/L
Bilirubin, total	7.6 mg/dL	0.0-1.5 mg/dL
Bilirubin, direct	5.3 mg/dL	0.0-0.4 mg/dL
Alk phos	179 IU/L	20-130 IU/L
AST	899 unit/L	0-35 unit/L
ALT	127 unit/L	0-40 unit/L
Lactic acid	13.3 mmol/L	0.5-2.0 mmol/L

**Figure 3:**
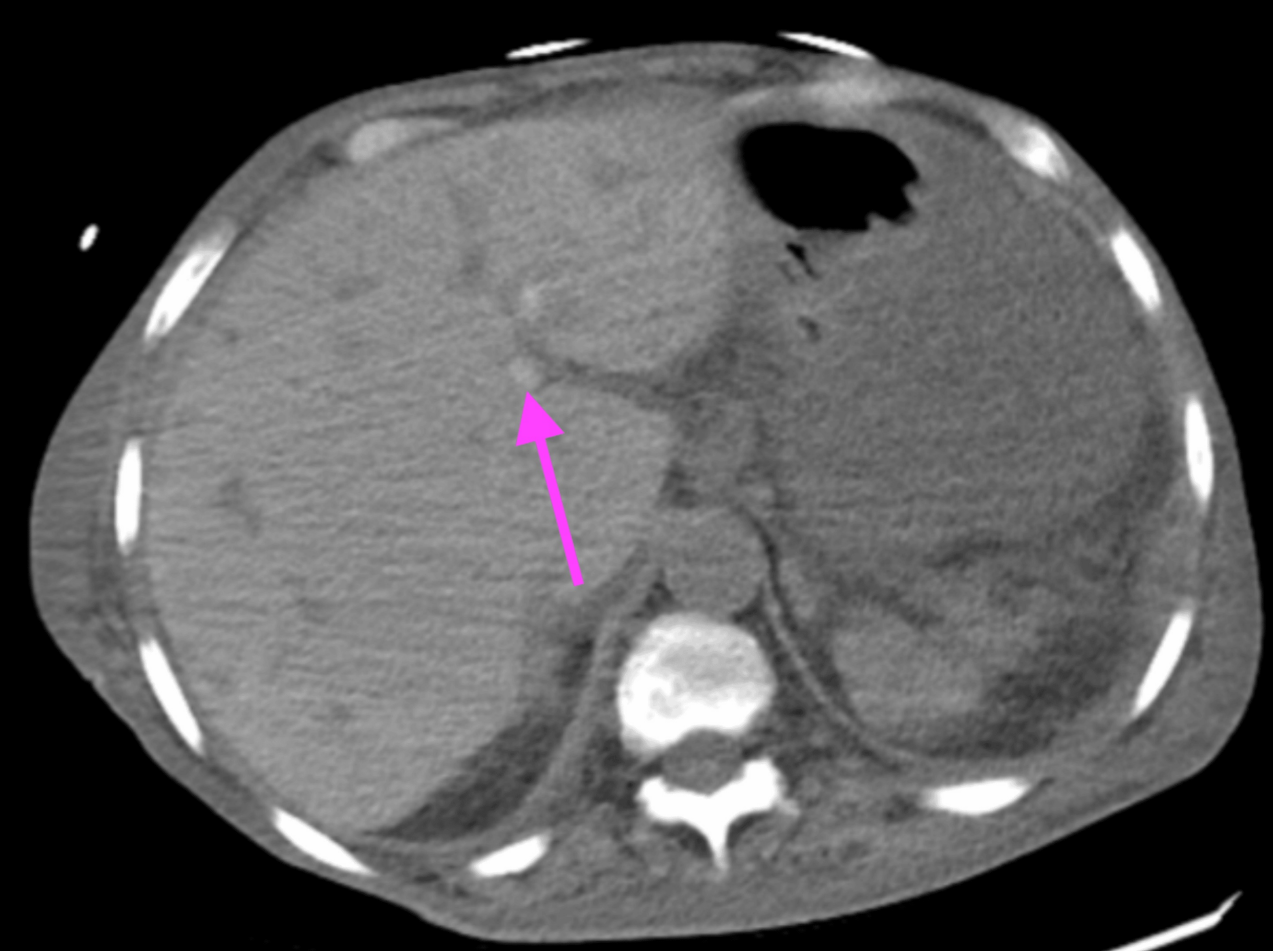
A CT scan of the abdomen and pelvis without contrast illustrated extensive liver disease with diffuse heterogeneity concerning for potential diffuse metastatic disease, contracted gallbladder with cholelithiasis, and a large amount of ascites with loculated peritoneal fluid collection in the lower portion of the abdomen and pelvis. The arrow depicts the heterogeneity of the liver.

The patient was admitted to the ICU for septic shock and subsequently intubated for airway protection. Two units of packed red blood cells were ordered; however, due to a history of multiple blood transfusions, she was found to have multiple autoantibodies, including anti-Vw, anti-E, anti-Fyb, anti-jkb, anti-K, anti-Lua, and anti-S. She was initiated on norepinephrine and a stress dose of dexamethasone 100 mg IV. Empiric vancomycin and meropenem 500 mg IV were started for her sepsis. The patient was also found to have refractory hyperkalemia with the initiation of continuous renal replacement therapy by nephrology. On the CT scan of the abdomen and pelvis, she was found to have extensive liver disease with diffuse heterogeneity concerning for potential diffuse metastatic disease, a contracted gallbladder with cholelithiasis, and a large amount of ascites with loculated peritoneal fluid collection in the lower portion of the abdomen and pelvis, as seen in Figure 3.

**Figure 4:**
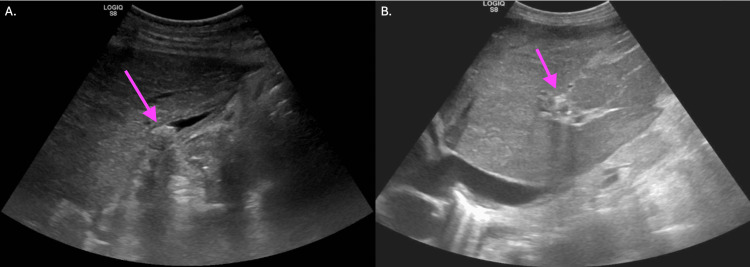
Ultrasound of the abdomen 4A shows an abdominal ultrasound showing no presence of liver masses, with gallstones and a decompressed gallbladder. The arrow indicates gallstones. 4B shows a repeat abdominal ultrasound illustrating a contracted gallbladder with gallstones within the gallbladder, a mildly dilated common bile duct, and a heterogeneous liver. The arrow highlights the heterogeneous liver.

Ultrasound of the abdomen showed no presence of liver masses, with gallstones and a decompressed gallbladder, as seen in Figure 4A.

The patient's bilirubin and liver function tests continued to rise throughout her stay. Figure 4B illustrates a repeat abdominal ultrasound, which was performed, resulting in a contracted gallbladder with gallstones within the gallbladder, a mildly dilated common bile duct, and a heterogeneous liver.

**Figure 5:**
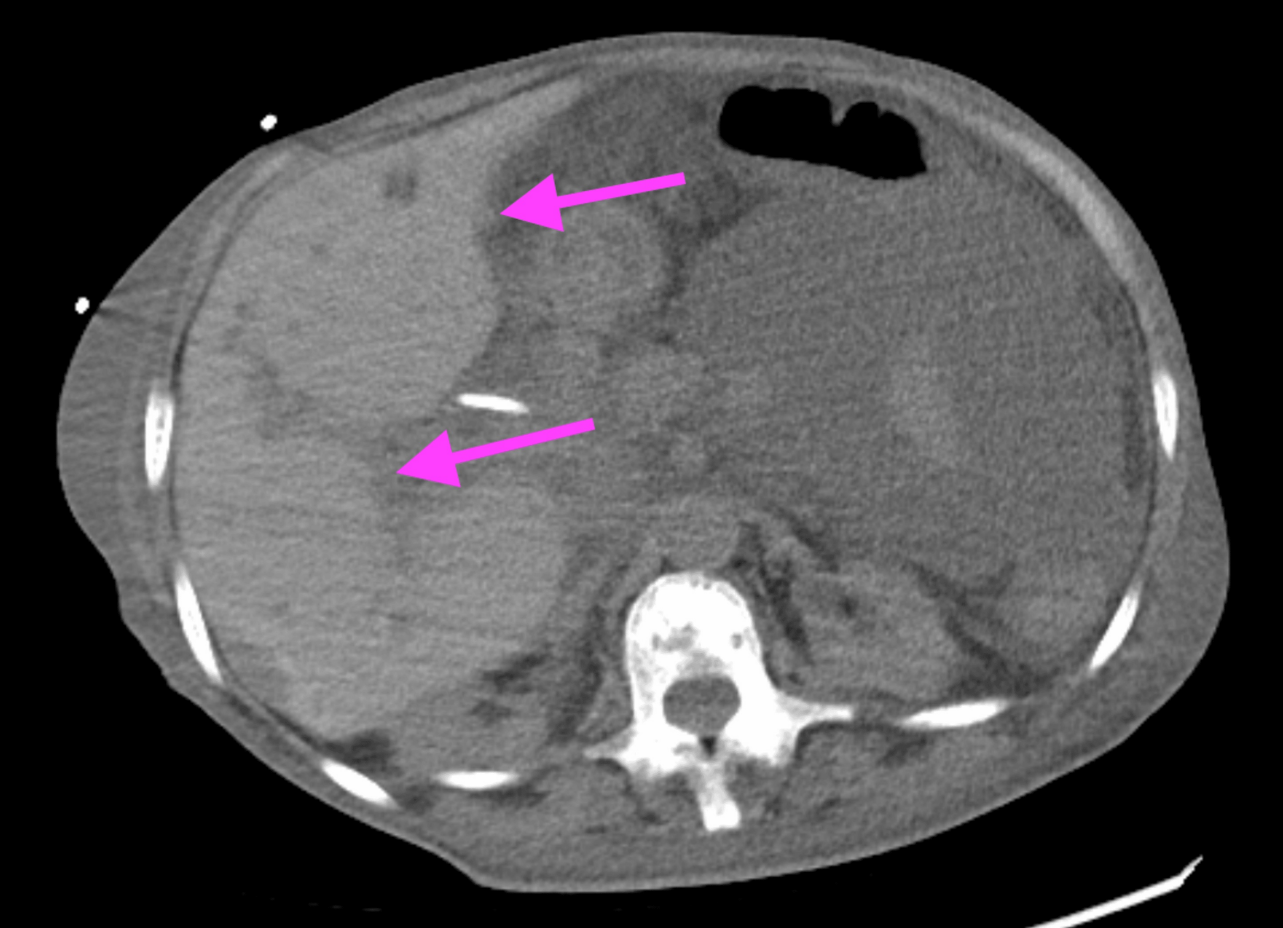
The final CT of the abdomen and pelvis showed a scalloping contour of the liver, possibly a peritoneal mass, cholelithiasis, and a large amount of ascites with ascending colon wall thickening, suggesting colitis, with arrows highlighting the areas of scalloping.

Magnetic resonance cholangiopancreatography (MRCP) showed a diffusely heterogeneous liver with nodularity overlying the liver, suggesting metastatic disease with omental metastasis, gallstones, and a common bile duct measuring 6.7 mm in diameter. The patient was then transferred to a different institution for interventional endoscopy. At that institution, a repeat CT scan of the abdomen and pelvis was ordered, showing a scalloping contour of the liver possibly representing an adjacent peritoneal mass, cholelithiasis, and a large amount of ascites with ascending colon wall thickening suggesting colitis, as shown in Figure 5.

The patient was then transferred to a tertiary center where she was placed on comfort care prior to IR omental mass biopsy. The patient, unfortunately, passed away at this tertiary care center.

## Discussion

HbSß is a rare overlap between sickle cell disease and beta-thalassemia, and those with Sß0 have demonstrated a poor clinical course [5]. Hemoglobinopathies, such as Sß0, can result in surgical asplenia as part of treatment or hyposplenia as part of the natural disease course. Splenic hypofunction results from splenic infarctions, leading to autosplenectomy earlier in life or progressively worsening function, which can result later in life [7]. In beta0-thalassemia specifically, the absence of β-globin chains results in excessive unbound alpha globin chains, leading to premature death via peripheral hemolysis and ineffective erythrocytosis, resulting in splenomegaly and ultimately hyposplenia, due to increased clearance of damaged red blood cells from the bloodstream [10]. This decreased function places patients at risk of various infections, typically with encapsulated organisms, such as *Neisseria meningitidis*, *Streptococcus pneumoniae*, and *Haemophilus influenzae* type b [7]. While these infections can be more common in patients with Sß0, liver abscesses are uncommon manifestations in these patients. Liver abscess in HbSß is a rare complication due to various organisms, including *Fusobacterium necrophorum*, *Escherichia coli*, *Klebsiella* sp., and *Candida albicans*. Most reported cases have been documented in children and young adults with Sß0 [11]. Our patient was found to have recurrent liver abscesses after drainage placement, and she was found to have an *Enterococcus cloacae* complex in one of her cultures. Due to concern for resistant bacteria from chronic antibiotic use with her dysfunctional immune system, she was treated with meropenem throughout her multiple admissions.

While this patient had an increased risk for infection due to her functional hyposplenia, she still required multiple hospitalizations for liver abscesses despite appropriate management. She continued to have admissions for concerns about sepsis despite having an intrahepatic drain placed during her first admission. During her last hospitalization, she was found to have worsening hyperbilirubinemia with elevated liver enzymes. Through imaging, she was found to have cholangitis via endoscopic retrograde cholangiopancreatography (ERCP) and an omental mass concerning for metastatic malignancy. While this patient was unable to undergo a biopsy of this mass, omental masses can be related to a multitude of different etiologies; however, malignancy is important to rule out. One such tumor to rule out is gastrointestinal stromal tumors (GISTs) [12]. GISTs can arise anywhere in the GI system, but commonly arise from the stomach and small intestine, with 60% arising from the stomach and 30% from the jejunum and ileum [12]. Managing patients with Sß0 is challenging due to the functional hyposplenia predisposing this population to infection. This impaired immune system requires a high level of suspicion when a patient presents with signs of systemic infection and will likely require a broad spectrum of antibiotic treatment and source control due to these patients’ hyposplenia.

## Conclusions

Sß0 is a rare condition affecting a small number of sickle cell anemia patients in the United States and is associated with a worsened disease prognosis. These patients often have this dysfunction of the spleen due to surgical asplenia as part of treatment or functional hyposplenia as a result of decreased splenic function. Liver abscesses are difficult to treat and have been rarely reported in these patients, with most reported cases being in adolescents and young adults. Given this difficulty in treatment for this process, further research is warranted on patients with Sß0, as they are often at higher risk of infections. Furthermore, omental masses, as seen in our patient, can be a result of various etiologies, with malignancies such as GISTs being important to rule out. Although our patient did not undergo a biopsy for a definitive diagnosis, this possibility should be considered.
